# A Case of Intussusception in Pregnancy: An Unexpected Complication Years After Bariatric Surgery

**DOI:** 10.7759/cureus.72212

**Published:** 2024-10-23

**Authors:** Shirley Ziton, Nicholas K Lago, Allison Draper, Breanne Kothe, Mario Gomez

**Affiliations:** 1 General Surgery, Broward Health Medical Center, Fort Lauderdale, USA; 2 Internal Medicine, Hospital Corporation of America (HCA) Florida/University of Central Florida College of Medicine Consortium, Fort Walton Beach, USA

**Keywords:** bariatric surgery, complications of bariatric surgery, intussusception, intussusception after bariatric surgery, pregnancy after bariatric surgery, roux-en-y bypass, roux-en-y complication, small bowel resection, small bowel resection in pregnancy, surgical complications in pregnancy

## Abstract

We present a case of bowel obstruction secondary to intussusception in a pregnant patient with a remote history of Roux-en-Y bypass. This patient presented with a one-day history of abrupt onset epigastric abdominal pain with associated nausea, emesis, and intolerance to oral intake. She was taken for an exploratory laparotomy after imaging suggested an internal hernia and a mass consistent with an intussusception was identified and resected. The patient later underwent a scheduled cesarean section at 38 weeks. Though bariatric surgery has been shown to reduce the risk of various conditions in pregnancy, the risk of developing complications, such as intussusception, should be considered when counseling female patients interested in bariatric surgery who may wish to become pregnant in the future. Additionally, pregnant patients with a history of bariatric surgery who present with acute abdominal pain should receive an urgent medical and surgical evaluation to rule out ischemic internal hernia or intussusception.

## Introduction

Bariatric surgery is a treatment option for obesity known to improve long-term health outcomes [1]. In regard to pregnancy, undergoing bariatric surgery prior to conception has been shown to improve fertility and decrease the risk of developing various complications such as gestational diabetes, macrosomia, pregnancy-induced hypertension, and preeclampsia [1]. However, bariatric procedures, such as Roux-en-Y gastric bypass, come with their risks. During pregnancy in a patient who has previously undergone bariatric surgery, there are increased incidences of intrauterine growth restrictions [1]. Unfortunately, these patients also carry an increased risk of intestinal obstruction [1]. These obstructions are most commonly caused by internal hernias; however, several reports of bowel obstruction secondary to intussusception, a condition where a portion of the bowel telescopes into itself, have also been documented [1]. Intussusception quickly becomes a surgical emergency as the portion of the bowel affected becomes edematous, leading to compression of the vasculature and subsequent ischemia and necrosis [1]. We present a case of small bowel obstruction secondary to intussusception in a pregnant patient with a remote history of Roux-en-Y bypass that was managed surgically to outline and discuss the management of this rare but life-threatening complication. 

## Case presentation

A 37-year-old female, gravida 1 para 0 (G1P0), at 29 weeks gestation with a history of Roux-en-Y gastric bypass nine years prior, presented to the emergency department complaining of abrupt onset epigastric abdominal pain that persisted for one day with associated nausea, emesis, and intolerance to oral intake. The patient was reportedly passing flatus and having bowel movements at the time of presentation. A detailed physical exam was difficult to obtain due to the presence of the gravid uterus; however, the patient had tenderness in the bilateral upper quadrants of the abdomen. 

**Figure 1:**
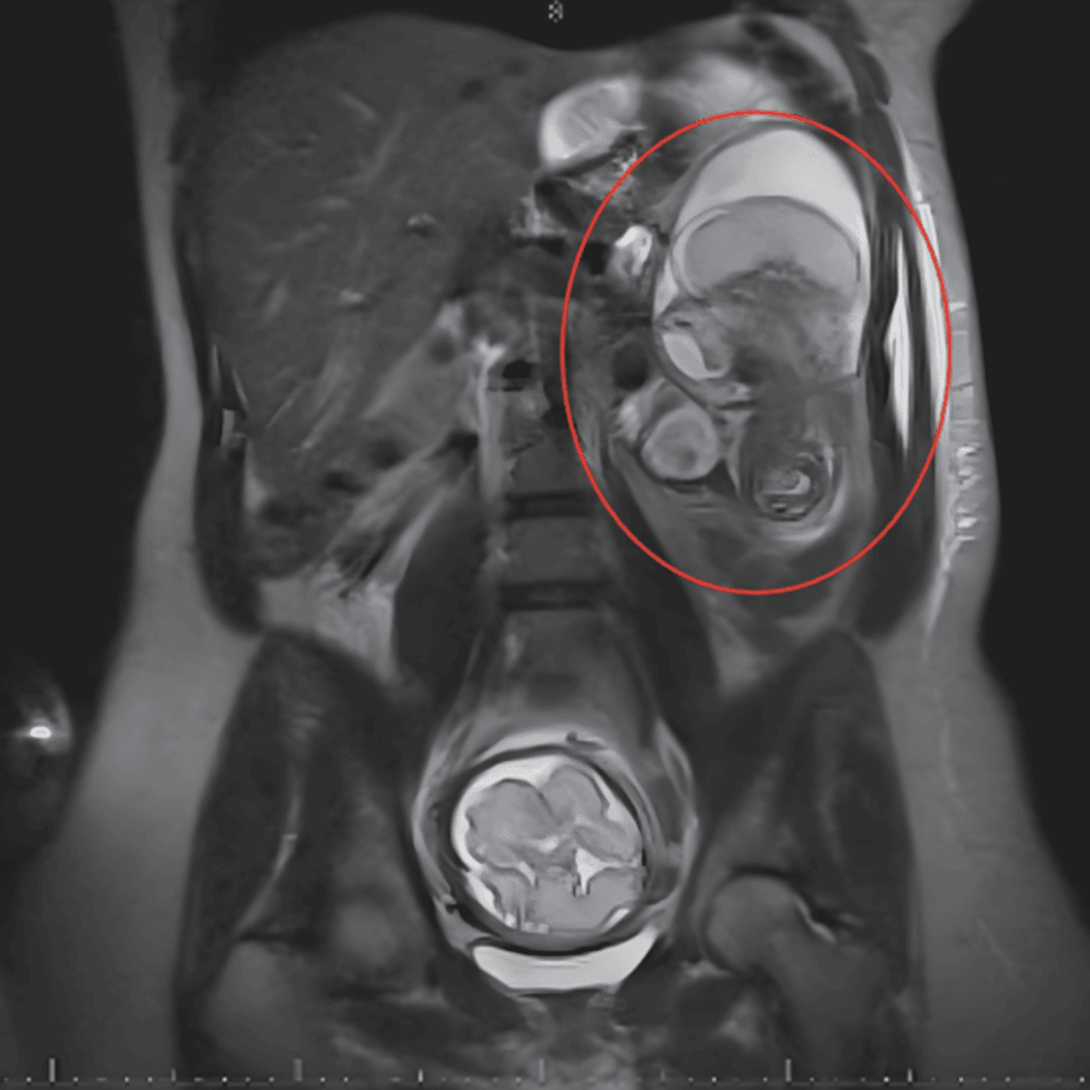
Abdominal MRI Magnetic resonance imaging (MRI) of the abdomen/pelvis of the 37-year-old female who was 29 weeks pregnant with symptoms consistent with a small bowel obstruction. Official read identified a closed-loop intestinal dilation suggesting an incarcerated internal hernia in the presence of a previous Roux-en-Y gastric bypass, suggesting a Petersen’s hernia.

Appendiceal, biliary, and obstetric pathologies were ruled out by ultrasound and lab values within normal limits. Endoscopy was significant only for mild gastritis. The patient’s clinical status continued to worsen, and she began complaining of worsening distension and lack of bowel movements. The decision was made to proceed with magnetic resonance imaging (MRI) of the abdomen and pelvis which revealed findings concerning for a Petersen’s hernia, as shown in Figure 1.

Therefore, the patient was taken for an exploratory laparotomy. The suspected Petersen’s hernia was located and manually reduced; however, this area did not appear grossly inflamed or edematous, so it was determined the hernia was not the cause of the patient's acute presentation. The bowel was then run, and a mass was located distal to the jejunojejunostomy, consistent with intussusception. This portion involved frankly necrotic bowel. 

**Figure 2:**
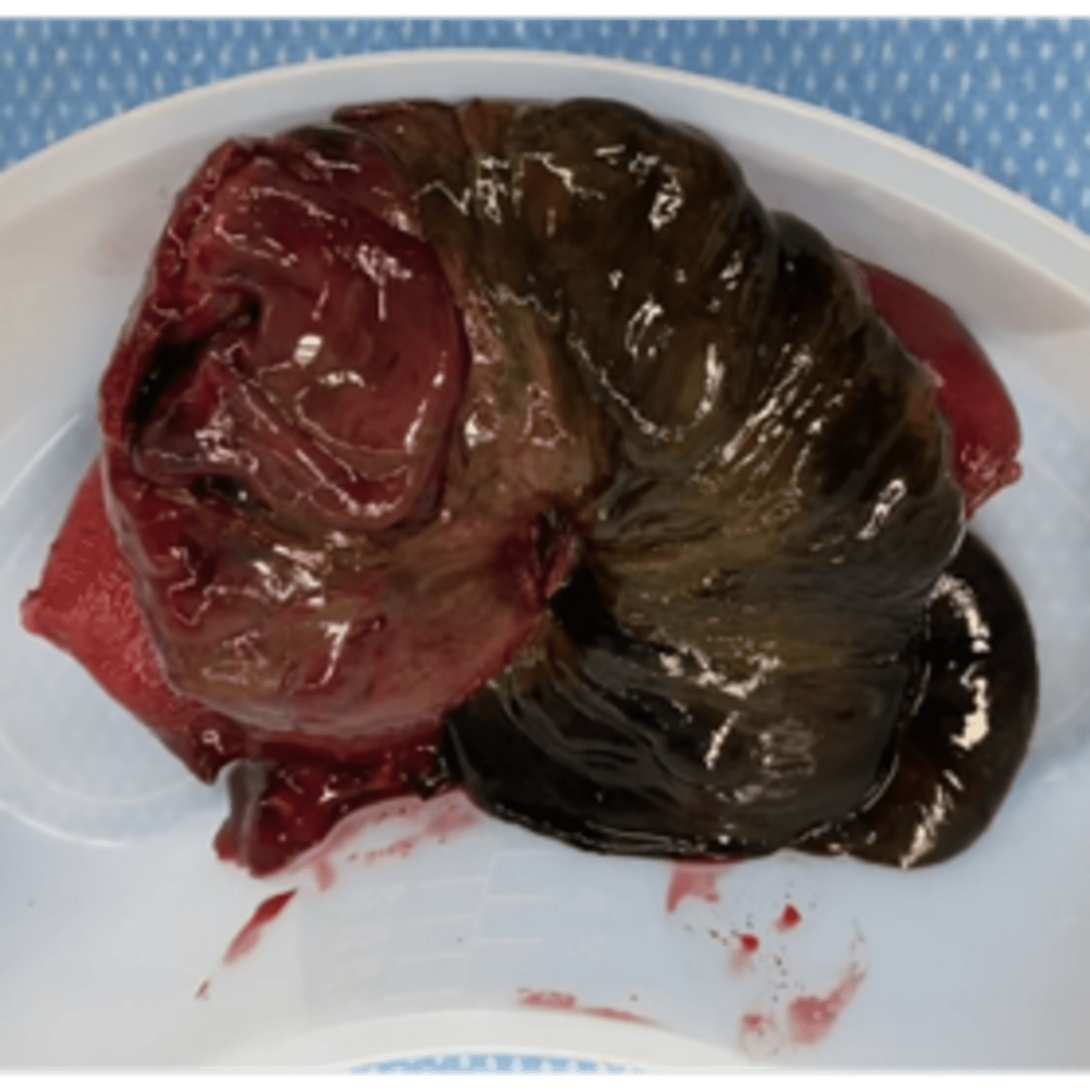
Surgical specimen Necrotic small bowel secondary to intussusception approximately 30 cm distal to the jejunojejunostomy in the patient with previous Roux-en-Y gastric bypass

This area of the small bowel was resected, as shown in Figure 2, and a side-side, functional, end-to-end anastomosis was performed. The small bowel was then run again to ensure no other obvious pathology; the abdomen was copiously irrigated and closed in a standard fashion. The patient clinically progressed, bowel function returned, and she was discharged tolerating a regular diet. Pathology from the resected section of the small bowel revealed focal ischemic necrosis with acute, inflamed, congested small bowel and no signs of malignancy or pathologic leadpoint. She underwent an uncomplicated, scheduled cesarean delivery at 38 weeks.

## Discussion

Intussusception typically occurs in an antegrade fashion as a result of peristaltic movement through a lead point caused by an adhesion or mass [1]. Interestingly, in the setting of Roux-en-Y bypass surgery, retrograde intussusception is more common, with the lead point originating from the jejunojejunostomy [1]. The etiology of intussusception after Roux-en-Y bypass is currently under debate; however, it is theorized that the creation of the roux limb disrupts the intestinal pacemakers of the small bowel, allowing for the formation of ectopic pacemakers in the Roux limb [1]. These pacemakers are theorized to generate electrical potentials in both proximal and distal directions, creating dysmotility, which creates a lead point for potential intussusception [1]. This explanation, though plausible, remains speculation at this time. 

Presentation of intussusception in pregnancy typically involves a myriad of non-specific symptoms, physical examination findings, and laboratory abnormalities, often resulting in delay of diagnosis and risk of clinical decompensation [1]. Due to the ambiguity of the clinical presentation in the present case, the diagnosis of intussusception and initiation of definitive treatment were delayed. Patients most frequently present complaining of acute-onset abdominal pain, nausea, and vomiting, as seen in this case [1]. 

Abdominal examination during pregnancy is often limited due to the presence of the gravid uterus, leading to a delay in diagnosis. This was a barrier to obtaining a thorough abdominal exam in the present case. Further, a computerized tomography (CT) scan is the diagnostic imaging study of choice for intussusception [2]. However, this imaging modality is avoided in pregnancy to prevent exposing the fetus to radiation, another factor that could contribute to delay in definitive diagnosis and treatment [3]. Magnetic resonance imaging, though not routinely used due to its increased examination time and cost, demonstrates similar sensitivity to CT and should be considered sooner in cases involving pregnant patients [2].

Once the diagnosis of intussusception is made, surgery should be initiated emergently to prevent progression to bowel ischemia and necrosis. Laparoscopic surgery is safe in pregnancy, and evidence has shown laparoscopic surgery offers the same benefit over open procedures when compared to non-pregnant patients [4-5]. Successful treatment has been documented with both resection and manual reduction; however, high rates of recurrence are reported with reduction [4]. In a case series of 23 patients requiring surgical intervention in the setting of intussusception, six patients were successfully treated with manual reduction, whereas 17 required resection [1], of those treated with resection, 65% presented with an ischemic segment [1]. This same case series reported only 26% of pregnant patients presenting with intussusception also delivered during the same hospital stay [1]. Only one case of fetal demise was documented, implying a minimal chance of poor fetal outcome following surgical intervention [1].

## Conclusions

Though bariatric surgery has been shown to reduce the risk of various conditions in pregnancy, including gestational diabetes and preeclampsia, the risk of developing complications, such as intussusception, should be considered when counseling female patients interested in gastric bypass surgery who may wish to become pregnant in the future. Further statistical analysis of the occurrence of bowel obstruction secondary to intussusception in pregnancy post Roux-en-Y gastric bypass and related outcomes would also offer clinicians further information regarding risk stratification for this patient population. Additionally, pregnant patients with a history of bariatric surgery who present with acute abdominal pain should receive an urgent medical and surgical evaluation to rule out ischemic internal hernia or intussusception. Bowel obstruction secondary to complications of previous bariatric surgery must be included in the differential for these patients, and further investigation, including tests such as MRI, should be considered early in these cases. Laparoscopic surgical intervention should be initiated emergently following diagnosis of intussusception and is proven to be a safe and potentially lifesaving treatment that poses minimal risk of poor maternal and fetal outcomes. Additionally, current literature lacks definitive evidence of the etiology of intussusception following Roux-en-Y gastric bypass and therefore remains an area necessitating future research.
